# Standardized pressure provocation as an objective clinical marker of omalizumab response in delayed pressure urticaria: an open-label, real-life, prospective cohort

**DOI:** 10.3389/fimmu.2026.1768128

**Published:** 2026-01-29

**Authors:** George N. Konstantinou, Indrashis Podder

**Affiliations:** 1 Department of Allergy and Clinical Immunology, 424 General Military Training Hospital, Thessaloniki, Greece; 2 Department of Dermatology, College of Medicine and Sagore Dutta Hospital, Kolkata, West Bengal, India

**Keywords:** atopy, autoimmune thyroid disease, chronic inducible urticaria, delayed pressure urticaria, dermographometer, omalizumab, pressure provocation test, physical urticaria

## Abstract

**Background:**

Delayed pressure urticaria (DPU) is a disabling chronic inducible urticaria characterized by delayed painful swelling after sustained pressure. Evidence-based therapeutic options remain limited, and omalizumab is frequently used off-label in antihistamine-refractory cases despite scarce prospective DPU-specific data with objective provocation testing.

**Methods:**

In a prospective, single-center, open-label cohort, adults with dermographometer-confirmed DPU refractory to licensed or high-dose second-generation H1-antihistamines received omalizumab 300 mg subcutaneously every 4 weeks. Patients were contacted daily during the first week after the initial dose and deliberately exposed to their typical pressure triggers to define time to complete symptom control. Baseline clinico-demographic characteristics, laboratory parameters, atopic status and comorbidities were recorded, and potential predictors of time to response were explored by univariate regression.

**Results:**

Forty-two patients were included (61.9% female; mean age at diagnosis 46.0 ± 14.0 years). Aeroallergen sensitization was present in 52.4% and clinically documented allergic disease in 26.2%; autoimmune thyroid disease was recorded in 33.3%. The DPU phenotype frequently involved soles and/or palms (54.8%), and angioedema (28.6%) and systemic symptoms (26.2%) were common. After the first omalizumab dose, 41/42 patients (97.6%) achieved complete symptom control within 96 hours (median 24 hours); 57.1% responded within 24 hours and 83.3% within 48 hours. One patient achieved only partial control. No serious adverse events or need for rescue systemic corticosteroids were observed. Time to response was not associated with sex, age, baseline total IgE, aeroallergen sensitization, atopic background, or autoimmune thyroid disease.

**Conclusion:**

In dermographometer-confirmed, antihistamine-refractory DPU, omalizumab was associated with a rapid clinical remission in almost all patients, typically within 24–48 hours, independent of baseline IgE and common comorbidities. Standardized pressure provocation testing provides objective confirmation of DPU and a pragmatic approach for diagnosis and real-world monitoring of therapeutic response in DPU. Given the open-label, uncontrolled design, these findings should be confirmed in larger multicenter controlled studies.

## 1 Introduction

Chronic urticaria is a mast cell–driven disease defined by the recurrence of wheals, angioedema, or both for more than 6 weeks and is classified into chronic spontaneous urticaria (CSU) and chronic inducible urticaria (CIndU), the latter characterized by reproducible eliciting triggers (1). Delayed pressure urticaria (DPU) is a CIndU subtype in which erythematous swellings and/or deep oedema occur at sites of sustained mechanical pressure after a characteristic time lag and can be painful and disabling (2). In classical descriptions, DPU lesions typically develop several hours after pressure, are often painful rather than pruritic, peak within hours, and resolve over 24–48 hours, although longer duration has been reported. DPU also frequently coexists with other chronic urticaria phenotypes. Early cohorts noted chronic urticaria in the majority of DPU patients, supporting overlap between spontaneous and pressure-induced whealing in real-life practice (3).

Beyond local swelling and functional impairment, systemic manifestations have long been recognized in DPU. In a seminal cohort, constitutional symptoms such as fever, chills and/or arthralgias were reported in a large proportion of patients, underscoring that DPU may extend beyond a purely cutaneous complaint (4). Subsequent clinical descriptions emphasized the substantial morbidity associated with painful pressure-site lesions, diagnostic delay, and frequent overlap with other urticaria patterns (3).

Accurate diagnosis of DPU is challenging in routine care because of the delayed onset of lesions after pressure exposure, which may obscure the trigger–response relationship, and because DPU can be present even in patients who do not spontaneously report pressure-related wheals (5). Accordingly, international consensus recommendations for CIndU emphasize provocation and threshold testing to confirm diagnosis, improve phenotypic precision, and enable objective monitoring of disease activity and treatment response (6). Dermographometer-based pressure challenge is a validated approach for DPU assessment. A 70-second dermographometer pressure challenge was shown to provide results comparable to weighted rod testing in chronic urticaria patients (5), and modified dermographometer protocols have been used to generate dose–response curves and to evaluate treatment effects in DPU (7).

Therapeutic management of DPU remains challenging. Importantly, no pharmacologic therapy is specifically licensed for DPU, and treatment approaches are largely extrapolated from chronic urticaria guidance and a limited, heterogeneous DPU−specific evidence base (8). In routine practice, licensed-dose second−generation H1−antihistamines (approved for urticaria) are used as first−line symptomatic therapy, together with trigger modification/avoidance; for patients who remain symptomatic, international urticaria guidelines allow up−dosing H1−antihistamines up to fourfold and short courses of systemic corticosteroids may be used as rescue for severe flares (1). Other off−label treatment options that have been reported for severe DPU include leukotriene receptor antagonists and immunomodulatory agents (e.g., ciclosporin), but the overall evidence remains limited and generally non−randomized (1, 8, 9).

While omalizumab is licensed for CSU (and recommended as add−on therapy for antihistamine−refractory CSU in current guidelines) (1, 10), its use in DPU is off−label. Recently, retrospective real-world analyses including patients with DPU have supported clinical effectiveness, although these often evaluate mixed CSU and DPU populations and do not fully resolve phenotype-specific predictors (11). Nevertheless, accumulating isolated DPU reports – from case reports to small series and DPU−focused real−world cohorts – support not only omalizumab clinically meaningful benefit in antihistamine−refractory DPU (11–14) but also rapid responses (13, 14).

In CSU, baseline total IgE and autoimmune endotype features have repeatedly been linked to differential response patterns, and low baseline IgE around the 43 IU/mL threshold has been proposed as a marker associated with autoimmune disease and type IIb autoimmune CSU characteristics (15). Autoimmune thyroid disease is also a well-established comorbidity in CSU, with systematic reviews demonstrating a strong association between CSU and thyroid autoimmunity (16), while chronic urticaria more broadly has been linked to an increased risk of autoimmune comorbidity (17). Whether these CSU-derived concepts translate to DPU – particularly in objectively confirmed DPU cohorts – remains unclear, as DPU-specific datasets have generally been small and heterogeneously defined (8, 14).

In addition, atopic background may be relevant in CIndU populations: atopy has been reported frequently in cold urticaria (18) and has been explored as a potential risk factor in symptomatic dermographism (19), but DPU-focused real-life profiling of atopy, laboratory patterns, and comorbidity burden remains limited.

Against this background and the limited DPU-specific real-world evidence, the objectives of the present study were to: (1) assess the effectiveness and safety of omalizumab in antihistamine-refractory DPU; (2) describe the clinico-demographic and laboratory profile of these patients, including allergic disease, atopy, autoimmune thyroid disease and broader comorbidity burden; and (3) explore potential clinical or laboratory predictors of time to treatment response in a real-life setting, with particular interest in variables that have been proposed as response modifiers in chronic urticaria (e.g., total IgE and autoimmune comorbidity) (15).

## 2 Materials and methods

### 2.1 Study design and setting

This was a prospective, single-center, open-label real-life cohort study conducted between 2013 and 2024 at the Department of Allergy and Clinical Immunology of a tertiary referral hospital in Greece. The study was approved by the Institutional Review Board (424 GMTH No: 12840) and conducted in accordance with the Declaration of Helsinki. Written informed consent was obtained from all participants.

### 2.2 Participants and diagnostic work-up

Adults presenting with clinical features suggestive of DPU were consecutively evaluated. Symptoms included delayed (typically 4–6 h) wheals or deep, often painful oedema at sites of sustained pressure (e.g., soles, palms, waistbands, shoulder straps, tight clothing).

**Figure 1:**
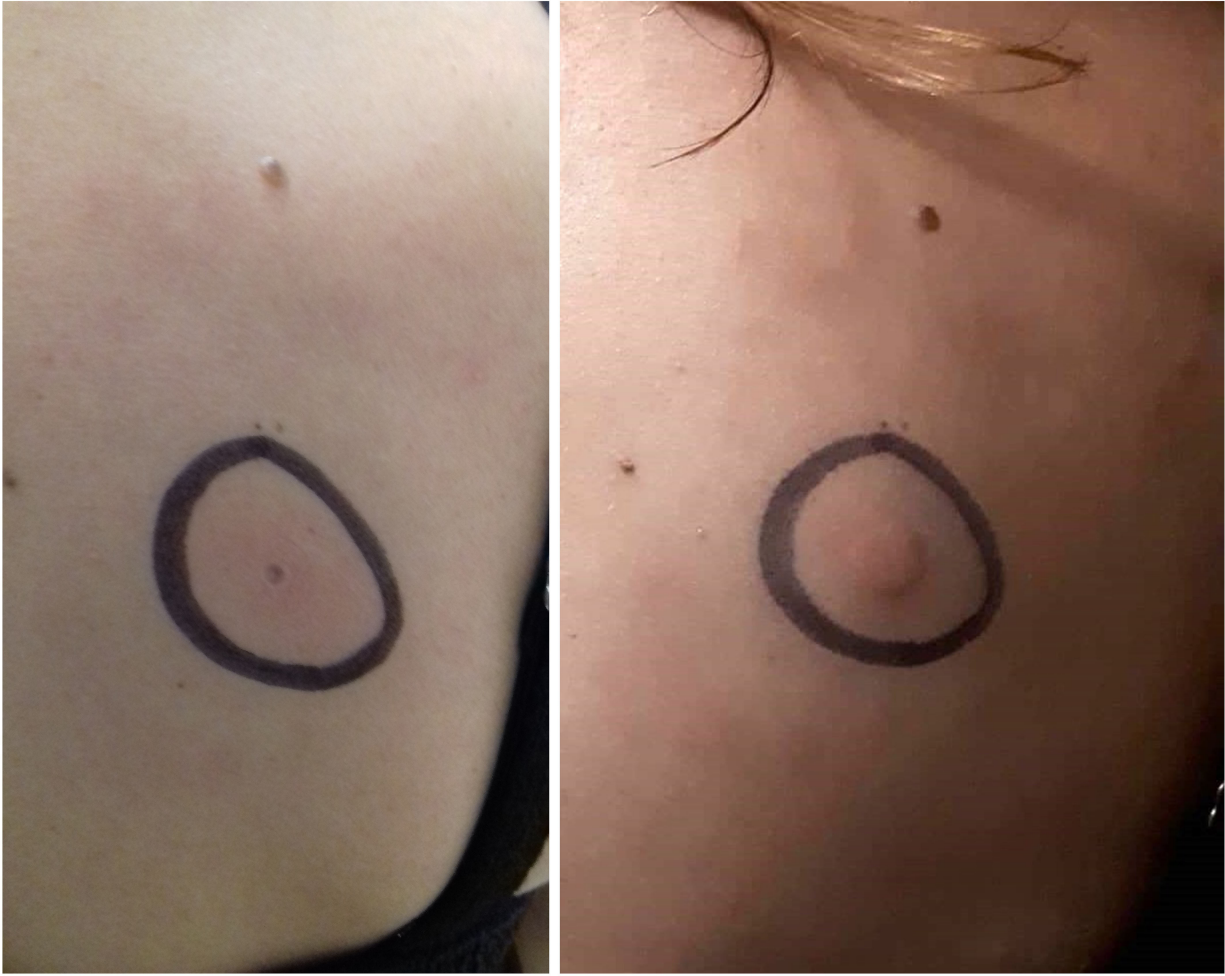
Dermographometer-based pressure provocation test confirming delayed pressure urticaria. Representative photographs of the standardized dermographometer pressure challenge site on the upper back. A calibrated dermographometer (100 g/mm²) was applied perpendicularly for 70 seconds. The application area was marked (black circle). The left panel shows the site immediately after provocation (baseline), and the right panel shows the delayed reading (6 hours later), demonstrating a palpable swelling consistent with a positive delayed pressure urticaria provocation test.

To confirm the diagnosis, a standardized pressure provocation test was performed using a calibrated dermographometer (HTZ Limited, UK). Antihistamines were discontinued for at least 3–4 days before testing. The dermographometer (100 g/mm²) was applied perpendicularly for 70 seconds to the upper back (5). The marked test site was evaluated at 6 h and 12 h after provocation. Patients were instructed to photograph the area at both time-points and email the images to the study physician. The test was considered positive if a palpable swelling was present at either reading (**Figure 1**). Patients with a typical clinical history and a positive pressure test were classified as having DPU and were eligible for inclusion.

Patients were excluded if they met any of the following criteria: i) concomitant chronic spontaneous urticaria (CSU), ii) concomitant other chronic inducible urticaria subtypes (e.g., symptomatic dermographism, cold urticaria), iii) ongoing long-term systemic corticosteroid or other immunomodulatory therapy, iv) pregnancy or lactation, or v) known hypersensitivity to omalizumab or any of its excipients.

### 2.3 Clinical and laboratory assessment

At baseline, a detailed medical history was recorded, including age at symptom onset, age at DPU diagnosis, temporal relationship between pressure and symptom onset, distribution and character of lesions, history of angioedema, and the presence of CSU-like wheals at non-pressure sites. Personal and family history of atopy (allergic rhinitis, asthma, food allergy, atopic dermatitis (AD)] and other comorbidities (endocrine, cardiovascular, musculoskeletal, gastrointestinal and autoimmune disorders) were systematically documented from their medical records.

All patients underwent measurement of serum total IgE and specific IgE to common aeroallergens (house dust mites, environmental molds, cat and dog dander, and common pollens from grasses, trees, and weeds) using standard immunoassays. Atopy was defined as the presence of serum-specific IgE >0.7 IU/mL to at least one environmental allergen. This conservative threshold was selected *a priori* to reduce potential misclassification related to borderline low-level results and analytical/result variability. Clinically documented allergic disease (e.g., allergic rhinitis, asthma, atopic dermatitis, food/venom allergy) was recorded separately from the medical record. Thyroid function tests (TSH, free T4) and thyroid autoantibodies (anti-TPO, anti-TG) were obtained to screen for autoimmune thyroiditis (e.g., Hashimoto’s thyroiditis, Graves’ disease) or other thyroid pathology. Additional investigations (e.g., inflammatory markers, ANA, infectious or gastrointestinal work-up) were performed based on clinical judgement.

### 2.4 Treatment protocol and follow-up

All included patients were antihistamine-refractory, defined as persistent DPU symptoms despite up to four-fold licensed doses of second-generation H1-antihistamines. They were treated with add-on omalizumab 300 mg subcutaneously every 4 weeks, in line with contemporary CSU treatment guidelines (1). Oral methylprednisolone 8 mg (up to twice daily for 4 days) was prescribed as rescue medication for uncontrolled flares.

For the first week after the initial omalizumab administration, all patients were contacted once daily by telephone. During these calls, patients reported the presence/absence of DPU symptoms, the time at which they first noted a clear improvement, the time of complete symptom resolution, any use of rescue corticosteroids and any treatment-emergent adverse events. If no improvement was reported within 7 days, patients were instructed to contact the study team unless this interval overlapped with the scheduled 4-week follow-up visit. During this period, patients were explicitly encouraged not to avoid activities that they associated with DPU exacerbations. Instead, they were asked to deliberately expose themselves to such pressure triggers (e.g., prolonged walking or standing, tight shoes, belts, brassieres, underwear, carrying heavy bags) to test the robustness of the treatment response.

### 2.5 Outcomes

The primary outcome was time to clinical response, defined as the time elapsed (in hours) from the first omalizumab injection to complete resolution of DPU symptoms after deliberate exposure to pressure triggers.

Secondary outcomes included: i) safety and tolerability of omalizumab, ii) the clinico-demographic and laboratory profile of antihistamine-refractory DPU patients (age at onset and diagnosis, diagnostic delay, atopy, serum total IgE, thyroid autoimmunity, other comorbidities), and iii) identification of clinical or laboratory predictors of time to response (serum IgE, Hashimoto thyroiditis, atopy, sex at birth, age at diagnosis).

### 2.6 Statistical analysis

Descriptive statistics were used to summarize the demographic and clinical characteristics of the study population. Continuous variables were assessed for normality using the Shapiro–Wilk test. Normally distributed variables are presented as means with standard deviations (SD), whereas non-normally distributed variables are expressed as medians with interquartile ranges (IQR). Categorical variables are summarized as counts and percentages.

For the primary endpoint (time to complete clinical response), the distribution was inspected and summarized using appropriate measures of central tendency (mean or median) and dispersion. In exploratory analyses, univariate tests were used to evaluate associations between time to response and potential predictors, including sex at birth, age at diagnosis, baseline total IgE levels, atopy status, and the presence of Hashimoto thyroiditis. Variables with a p-value < 0.25 in univariate analysis were prespecified for inclusion in a multivariable regression model. All tests were two-sided, and p < 0.05 was considered statistically significant. Analyses were performed with Stata v10.0; graphs were produced using GraphPad Prism.

## 3 Results

### 3.1 Study population

**Figure 2:**
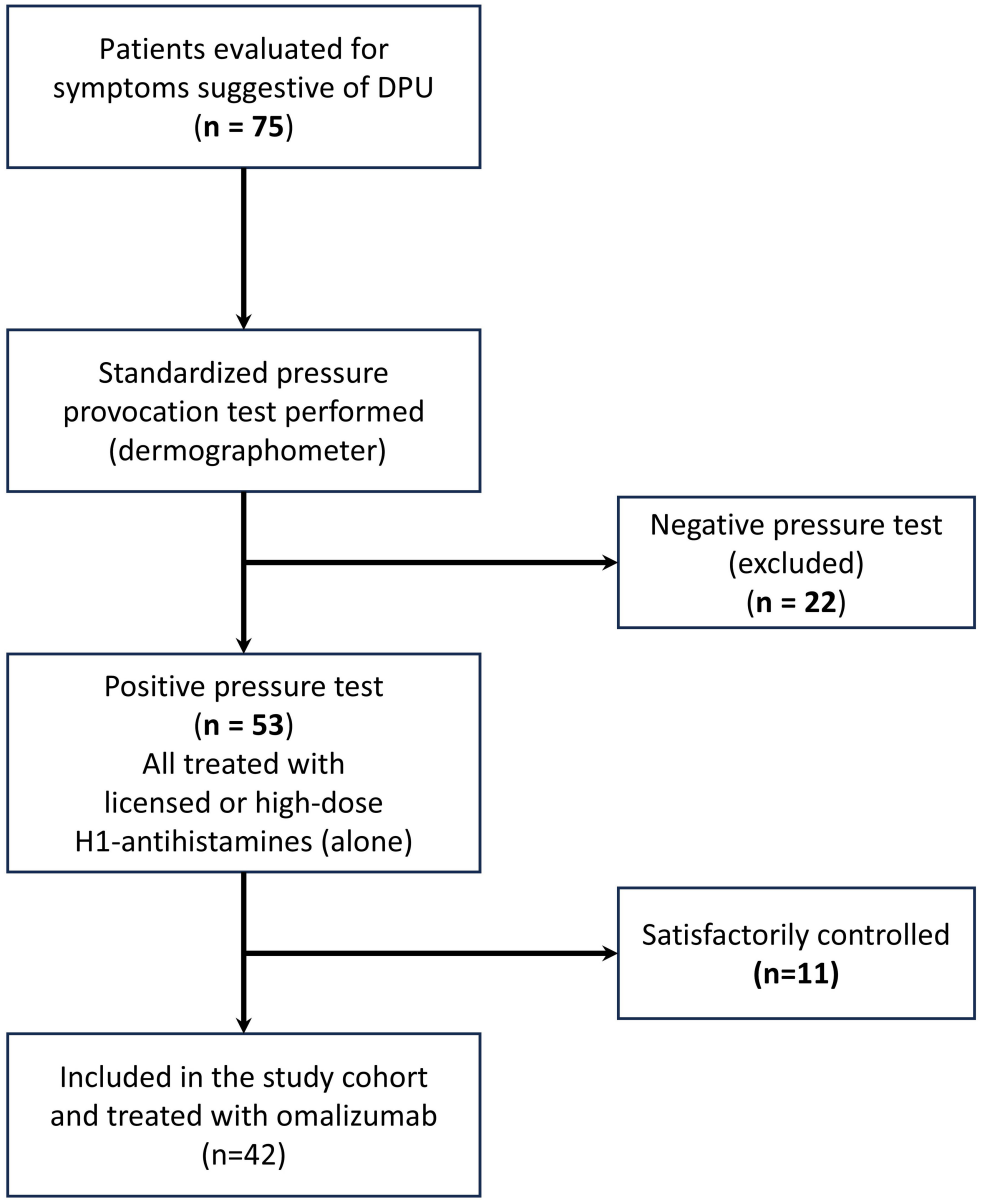
Study flow diagram and cohort selection. Between 2013 and 2024, 75 adults were evaluated for symptoms suggestive of delayed pressure urticaria (DPU) and underwent standardized dermographometer pressure provocation testing. Fifty−three patients had a positive pressure test. Of these, 11 achieved satisfactory symptom control with licensed or high−dose second−generation H1−antihistamines alone and did not require biologic therapy. The remaining 42 patients did not achieve adequate control with antihistamines, fulfilled eligibility criteria, were treated with omalizumab (300 mg subcutaneously every 4 weeks), and were included in the analysis.

Between 2013 and 2024, 75 patients were evaluated for symptoms suggestive of DPU. Fifty-three had a positive pressure test. Of these, 11 achieved satisfactory symptom control with licensed or high-dose second-generation antihistamines alone and did not require biologic therapy. The remaining 42 patients fulfilled all inclusion and exclusion criteria and were treated with omalizumab. These 42 patients constitute the study cohort. (**Figure 2**).

The cohort comprised 26 women (61.9%) and 16 men (38.1%), with a mean age at DPU onset of 40.2 ± 15.8 years (range: 11.5–75.2 years) and a mean age at diagnosis of 46.0 ± 14.0 years (range: 16.1–75.6 years). The interval between symptom onset and formal diagnosis was highly variable, ranging from almost immediate diagnosis (within a month) up to 27.7 years, with a median diagnostic delay of approximately 1.4 years (IQR 0.1-9.0 years).

The median serum total IgE level was 100.5 IU/mL (IQR 54.7–186.3 IU/mL), with values ranging from 15.3 to 1468 IU/mL, indicating marked heterogeneity in IgE levels within the cohort. Using the 43 IU/mL cut-off proposed as a biomarker of type IIb autoimmune CSU (15), 9/42 (21.4% of the total cohort) had low IgE levels (≤ 43 IU/mL).

Allergen sensitization data (serum−specific IgE to common aeroallergens) showed that 22/42 patients (52.4%) were sensitized to at least one aeroallergen, most frequently grass and Bermuda grass pollens, house dust mites, and cypress or olive tree pollens. Occasional sensitization to other allergens (e.g., *Parietaria judaica*, *Alternaria alternata*) was also observed. Based on the clinical records, 11/42 patients (26.2%) had known allergic diseases: four isolated allergic rhinitis, two allergic rhinitis and asthma, one allergic rhinitis and atopic dermatitis, two allergic rhinitis, asthma and AD, one food allergy to sesame and one allergy to yellow jacket venom.

Autoimmune thyroiditis was recorded in 14/42 patients (33.3%) (13/42 with Hashimoto’s thyroiditis and 1/42 with Graves’ disease). Quantitative antibody titers were not consistently available for the entire cohort and were therefore not analyzed.

Approximately 38% (16/42) had at least one additional chronic comorbidity documented in their medical record, most commonly endocrine (autoimmune thyroid disease, hyperparathyroidism), cardiovascular (arterial hypertension), metabolic (obesity, diabetes), gastrointestinal (gastro-esophageal reflux, gastritis, irritable bowel syndrome) and urogenital conditions (prostatitis). **Table 1** summarizes the baseline cohort demographics.

**Table 1:** Baseline cohort characteristics.

Characteristic	Total cohort (n = 42)
Sex, female	26 (61.9%)
Age at DPU onset (years), mean ± SD	40.2 ± 15.8
Age at DPU diagnosis (years), mean ± SD	46.0 ± 14.0
Diagnostic delay (years), median (IQR)	1.4 (0.1–9.0)
Total IgE (IU/mL), median (IQR)	100.5 (54.7–186.3)
range (IU/mL)	15.3–1468
total IgE ≤43 IU/mL	9/42 (21.4%)
Aeroallergen sensitization	22 (52.4%)
Any clinically documented allergic disease*	11 (26.2%)
Allergic rhinitis	9 (21.4%)
Asthma	4 (9.5%)
Atopic dermatitis	3 (7.1%)
Food allergy	1 (2.4%)
Yellow-jacket venom allergy	1 (2.4%)
Autoimmune thyroid disease	14 (33.3%)
Hashimoto’s thyroiditis	13 (31.0%)
Graves’ disease	1 (2.4%)
Any additional chronic comorbidity^‡^	16 (38.1%)

* Allergic conditions are not mutually exclusive (patients may have >1).

‡ As recorded in the medical chart (examples reported include endocrine, cardiovascular, metabolic, gastrointestinal and urogenital comorbidities).

Abbreviations: DPU, delayed pressure urticaria; SD, standard deviation; IQR, interquartile range; IgE, immunoglobulin E.

### 3.2 Clinical phenotype of DPU flares

Detailed narrative histories were reviewed to better characterize symptom patterns and triggers of DPU flares. Across these histories, the clinical phenotype of DPU was remarkably stereotyped and pressure-driven (**Table 2**). More than half of patients (23/42, 54.8%) described wheals or deep oedema on the soles and/or palms, often as a very early or even initial manifestation of disease, and frequently severe enough to interfere with walking or manual activities.

**Table 2:** Delayed pressure urticaria-related clinical phenotype, distribution, and trigger contexts in the study cohort (n = 42).

Clinical features	n (%)
Soles and/or palms involvement	23 (54.8%)
Angioedema (face/lips/extremities) during flares	12 (28.6%)
Systemic symptoms accompanying lesions	11 (26.2%)
Psychological stress/emotional strain reported as aggravating factor	12 (28.6%)
Nocturnal awakening due to flares	5 (11.9%)
Burning pain predominant (rather than pruritus)	5 (11.9%)
Buttock lesions linked to prolonged sitting (office work)	2 (4.8%)
Forearm/elbow plaques at desk contact points (keyboard use)	2 (4.8%)
Occipital scalp swelling triggered by traction hairstyle (tight ponytail/bun)	3 (7.1%)
Perineal swelling/dyspareunia triggered by sexual intercourse	1 (2.4%)

Angioedema of the face, lips or extremities accompanied flares in roughly one-third of patients (12/42, 28.6%). Systemic symptoms, most commonly fatigue, musculoskeletal pain, or flu-like malaise accompanying DPU lesions, were reported by 11/42 patients (26.2%).

Psychological stress or emotional strain was explicitly mentioned as an aggravating factor by 12/42 patients (28.6%), often together with physical triggers such as prolonged walking or standing, tight shoes or belts, brassieres or underwear. In two further patients, buttock lesions were clearly linked to prolonged sitting during office work, and in a further two patients, plaques on the elbows or forearms developed exactly at the contact points with the desk surface during keyboard use. In addition to classical weight-bearing areas, three women described recurrent, tender swellings over the occipital scalp exactly at the site where a tight ponytail or bun was pulling on the skin, providing another highly suggestive, pressure-related clue to DPU involvement of the scalp. One previously published patient from this cohort experienced deep perineal swelling triggered by sexual intercourse, leading to dyspareunia (20).

In a smaller subset, DPU flares were described as sufficiently intense to cause nocturnal awakening (5/42, 11.9%) or to be dominated by burning pain rather than pruritus (5/42, 11.9%), with one patient characterizing the symptoms as “very severe pain”.

### 3.3 Response to omalizumab and safety

**Figure 3:**
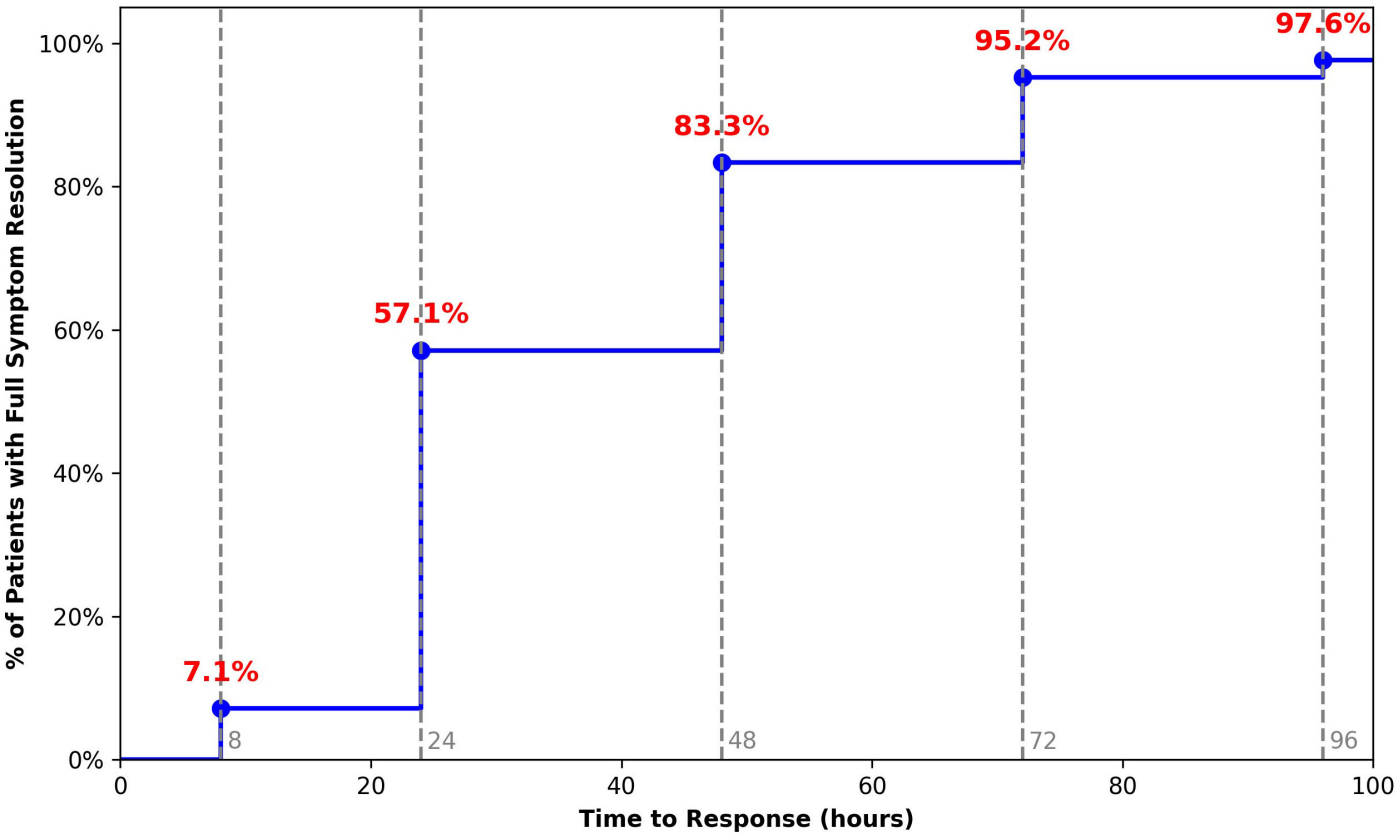
Rapid onset of complete symptom control after the first omalizumab dose in antihistamine-refractory delayed pressure urticaria. Cumulative time-to-response curve showing the proportion of patients achieving complete delayed pressure urticaria symptom resolution after the first omalizumab administration (300 mg subcutaneously). Response time was defined as the elapsed time (hours) from the first injection to complete resolution of DPU symptoms after deliberate exposure to typical pressure triggers. One patient achieved only partial symptom control and is therefore not included among the complete responders, explaining the plateau at 97.6%. Vertical dashed lines indicate the prespecified assessment timepoints used to derive the cumulative response proportions.

All 42 patients were treated with omalizumab 300 mg every 4 weeks on top of background antihistamines. Among complete responders (41/42), time to complete symptom resolution after the first injection ranged from 8 to 96 hours, with a median of 24 hours (IQR 24-48). Three patients (7.1%) reported complete remission of DPU symptoms within 8 hours of the first dose. By 24 hours, 24/42 (57.1%) were symptom-free, 35/42 patients (83.3%) achieved complete resolution within 48 hours, and 40/42 (95.2%) within 72 hours. One patient experienced complete resolution by 96 hours, and another one had only a partial response with persistent, though markedly attenuated, symptoms at 168 hours. The cumulative time-to-response curve is shown in **Figure 3**, illustrating the steep early rise in the proportion of patients achieving complete control within the first 2–3 days after omalizumab initiation.

Omalizumab was very well tolerated. No patient reported serious or unexpected treatment-emergent adverse events and none required systemic rescue corticosteroids.

### 3.4 Predictors of time to response

In univariate linear regression analyses, no baseline demographic, clinical, or laboratory variables were associated with time to complete response (including serum total IgE, Hashimoto thyroiditis, atopy, sex at birth, or age at diagnosis) As no variable met the pre-specified p < 0.25 threshold, (IgE p=0.57, Hashimoto thyroiditis p=0.82, atopy p=0.75, sex at birth p=0.64, age at diagnosis p=0.81) multivariable regression analysis was not performed.

## 4 Discussion

Delayed pressure urticaria is a chronic inducible urticaria (CIndU) phenotype characterized by delayed, frequently painful wheals and/or deep oedema that develop hours after sustained mechanical pressure and can persist for many hours to several days (2, 3, 21). Because symptoms occur with a time lag and at mechanically stressed sites, DPU is often under-recognized, misattributed to infection or “idiopathic swelling”, and diagnosed late unless pressure-triggered symptoms are actively explored and provocation testing is performed (2, 5). The burden of disease can be profound, as DPU preferentially affects weight-bearing and load-bearing areas (feet, hands, waist/shoulder strap zones), compromises occupational performance and mobility, and is commonly associated with impaired quality of life (3, 21–23). Importantly, systemic manifestations such as malaise, flu-like symptoms, fever/chills and arthralgias have been described in a substantial subset of patients, emphasizing that DPU is not always a purely cutaneous disorder (3, 4).

In this context, the present prospective real-life cohort provides several clinically actionable insights. First, omalizumab demonstrated rapid, robust and clinically striking effectiveness in antihistamine-refractory, dermographometer-confirmed DPU, with most patients achieving complete control within 24–48 hours after the first 300 mg dose. This ultra−rapid response phenotype is consistent with the broader CIndU omalizumab literature, in which rapid onset (sometimes within 24 hours) has been repeatedly noted, albeit mostly in smaller datasets and across heterogeneous inducible subtypes (24, 25). For DPU specifically, published evidence has historically been limited to case reports and small series, which nevertheless converge on the notion that omalizumab can induce rapid and meaningful disease control in otherwise treatment-resistant patients (12–14, 26, 27). More recent tertiary-care experiences have also used pressure-provocation testing as an objective outcome measure and reported elimination of provocation-induced wheals after omalizumab exposure, supporting a true biological effect rather than avoidance behavior alone (11). Taken together with guideline-based stepwise management, where omalizumab is recommended as add-on therapy for antihistamine-refractory chronic urticaria and increasingly applied to CIndU, albeit often off-label, our findings support consideration of omalizumab as an escalation option in patients with objectively confirmed, antihistamine-refractory DPU (1, 6). However, given the observational, uncontrolled design and single-center setting, comparative effectiveness and optimal sequencing require confirmation in controlled studies.

Second, our study shows that the standardized dermographometer pressure provocation test is not just a diagnostic tool but also a practical clinical indicator that defines the phenotype and could significantly improve the identification of omalizumab-responsive DPU in real-world settings. Provocation and threshold testing are explicitly recommended in international consensus documents for CIndU to confirm diagnosis and to quantify trigger thresholds, thereby improving diagnostic certainty and enabling objective monitoring (6, 28). DPU is particularly prone to diagnostic ambiguity because delayed lesions can mimic other inflammatory swellings, and a subset of chronic urticaria patients may have positive pressure challenges even when pressure is not spontaneously recognized as a trigger (5). In our cohort, the requirement for both a typical clinical history and a positive dermographometer test likely reduced phenotype contamination (e.g., misclassified CSU or other inducible subtypes), which may help explain the consistency and magnitude of response. In this sense, a positive dermographometer-based pressure test functions analogously to a “functional clinical biomarker”. It is an objective, stimulus-linked readout of disease biology that strengthens treatment selection and allows mechanistically meaningful assessment before and after therapy (6, 11, 27). However, the proposition that a positive pressure test is the only predictor of omalizumab response “in urticaria” requires qualification. In CSU, several biomarkers and endotype-associated features (particularly baseline total IgE and markers of type IIb autoimmune CSU) have been repeatedly linked to likelihood and speed of omalizumab response (15, 29–31). Thus, rather than being a unique predictor across all urticaria, pressure provocation positivity may be best conceptualized as the most practical and reproducible objective indicator currently available for DPU case definition and response monitoring, in a field where validated laboratory predictors remain sparse for this specific phenotype.

Third, the exceptionally rapid onset of omalizumab effect observed in our cohort is biologically plausible and informative. Omalizumab binds circulating IgE, reduces free IgE, and leads to downregulation of FcϵRI on mast cells and basophils—mechanisms that can translate into relatively rapid suppression of mast-cell reactivity in susceptible patients (32, 33). While DPU pathophysiology is incompletely defined, lesional studies support a prominent inflammatory mediator milieu, including upregulation of TNF-α and IL-3 in urticaria skin (34), and a potential link between systemic symptoms and IL-1-driven acute-phase biology in severe DPU (35, 36). Histologic and immunopathologic work also implicates eosinophil activation and granule protein deposition in DPU lesions, particularly in bullous variants, reinforcing that DPU may involve more than histamine alone (36–38). Therapeutic responsiveness to anti–TNF-α in severe DPU further supports a role for TNF-α-dependent inflammation in at least some patients (39). Against this mechanistic background, our observation that a single omalizumab dose can abolish clinically meaningful pressure-induced disease within 1–2 days in most patients suggests that IgE/FcϵRI-dependent pathways may be central to the “trigger-to-wheal” cascade in a large subset of DPU patients, even if downstream inflammatory amplification (including eosinophilic components) contributes to lesion persistence and pain in severe phenotypes (21, 40).

Fourth, our data support the notion that omalizumab benefits in DPU are largely independent of baseline demographic and clinical characteristics, including age, sex at birth, atopic background, autoimmune thyroiditis, and total IgE. In exploratory regression analyses, none of these variables showed even a trend-level association with time to complete response, and therefore, multivariable modeling was not pursued. This is clinically important. It implies that clinicians should not delay escalation to omalizumab in objectively confirmed, antihistamine-refractory DPU based on baseline IgE level or the presence of common comorbidities. This also aligns with published DPU omalizumab experiences that describe effectiveness across diverse patient profiles, although prior datasets were smaller and often lacked systematic early kinetics (13, 14). More broadly, systematic reviews of omalizumab across CIndU subtypes support substantial benefit with rapid symptom control in many cases, even though controlled trials are still limited for several inducible phenotypes (24, 25).

Fifth, our cohort offers a useful perspective on total IgE in DPU relative to CSU. In CSU, low baseline total IgE (often operationalized around 43 IU/mL) has been proposed as a marker of the type IIb autoimmune endotype and has been associated in multiple studies and meta-analyses with reduced likelihood or slower kinetics of response to omalizumab (15, 29, 30). In the present DPU cohort, a meaningful proportion of patients met this “low IgE” criterion, yet responses remained rapid and robust overall, arguing that baseline total IgE is not a clinically useful discriminator of omalizumab responsiveness once the DPU phenotype is objectively confirmed. One possible interpretation is that DPU encompasses mechanistic heterogeneity, and that IgE-independent autoimmune activation (prominent in type IIb CSU) may not be the dominant driver in many DPU patients, even when serum IgE levels are low. Alternatively, omalizumab may exert clinically relevant effects in DPU through mechanisms that are less tightly coupled to baseline IgE concentration than in CSU, such as modulation of mast-cell FcϵRI expression dynamics or downstream cellular activation thresholds (33, 40). These hypotheses merit direct mechanistic testing in DPU, where immunophenotyping studies remain comparatively scarce (21).

Sixth, autoimmune thyroiditis was common in our cohort, affecting approximately one-third of patients. Thyroid autoimmunity is a well-established comorbidity in CSU. Systematic reviews document increased prevalence of antithyroid antibodies and thyroid dysfunction in CSU compared with controls, with Hashimoto’s thyroiditis and hypothyroidism being more common than Graves’ disease (16). More broadly, CSU is associated with a spectrum of autoimmune comorbidities, supporting an “autoimmunity-prone” background in a substantial subset of chronic urticaria patients (17, 30). Although our cohort was defined by DPU rather than CSU, the thyroiditis prevalence may reflect shared immunogenetic susceptibility across urticaria endotypes and/or partial overlap of inducible and spontaneous whealing biology, a phenomenon repeatedly noted across chronic urticaria phenotypes (41, 42). Clinically, our findings suggest that autoimmune thyroiditis should be viewed as a common co-traveling condition rather than a negative prognostic marker for early omalizumab response in DPU.

Seventh, we observed a substantial atopic signal in this DPU cohort, both in terms of aeroallergen sensitization and clinically documented allergic disease. Evidence for atopy as a predisposing factor is particularly established in other inducible urticarias. For instance, classic data in cold urticaria demonstrate frequent atopy and higher total IgE levels in affected patients (18), and more recent symptomatic dermographism datasets support higher atopy prevalence as well (19). While DPU-specific atopy epidemiology is less extensively characterized, our findings are biologically plausible given the central role of mast cells and IgE/FcϵRI biology across multiple urticaria phenotypes (6, 40). From a practical standpoint, the observed atopic background reinforces the concept that DPU patients seen in tertiary settings may represent an “atopic-like” mixed immunologic landscape rather than a purely autoimmune subset, underscoring the need for objective phenotyping (provocation testing) and individualized management strategies.

Eighth, the comorbidity burden in our cohort was notable. Several factors may explain this. Firstly, chronic urticaria is itself linked to increased rates of autoimmune conditions and other chronic diseases, likely reflecting shared immune dysregulation and systemic inflammatory crosstalk (17, 30). Secondly, severe and painful DPU can drive repeated healthcare encounters and extensive diagnostic work-ups, increasing the likelihood of identifying coincident comorbidities (2, 3). Thirdly, DPU is associated with systemic symptoms in a subset of patients and can display inflammatory laboratory signatures in severe/bullous cases, supporting a broader systemic inflammatory component that may relate to comorbidity clustering in at least some individuals (4, 35). Finally, referral bias is likely. Tertiary cohorts may over-represent severe, long-standing, multimorbid patients whose disease is refractory to antihistamines and therefore escalated to biologic therapy.

When placed against the backdrop of historical DPU cohorts, our clinical observations also resonate with classical descriptions while adding contemporary granularity. Dover et al. colleagues reported substantial disability, prolonged disease duration, and high rates of systemic flu-like symptoms, highlighting DPU severity and heterogeneity (3). Sussman et al. described painful lesions that peak approximately nine hours after onset and resolve within 24 to 48 hours, with systemic symptoms observed in the majority of patients (4). Our cohort similarly emphasized pain, functional impairment and occasional systemic symptoms, but also captured highly specific, mechanistically feasible, pressure-point distributions that may be under-reported in routine histories (e.g., buttock lesions from prolonged sitting, elbow/forearm plaques at keyboard/desk contact points, occipital scalp swelling from traction hairstyles). These observations reinforce a key diagnostic principle: DPU triggers can be subtle and embedded in modern daily behaviors, and meticulous trigger mapping can reveal highly suggestive patterns that should prompt formal provocation testing (6). The previously published dyspareunia case from this cohort further extends the clinical spectrum of pressure-triggered swelling and underscores that DPU can involve sites not typically considered in standard physical urticaria histories (20). Additional reports also highlight sexual intercourse as a trigger for inducible urticaria phenotypes, supporting broader clinical awareness in this domain (43).

Several strengths enhance the interpretability and potential impact of our findings. This cohort is, to our knowledge, the largest series of prospectively characterized, provocation-confirmed DPU patients treated with a standardized omalizumab regimen. It is also the first to systematically capture response kinetics at an hour-level resolution during the early post-dose period. In contrast, prior omalizumab evidence in DPU has largely been limited to individual cases or small case series (12–14, 27). Focusing on objective provocation positivity aligns with international CIndU guidelines and enhances causal inference by minimizing phenotype misclassification (6). A further pragmatic strength is that patients were explicitly encouraged to challenge themselves with real-life pressure triggers during early follow-up, reducing the likelihood that the apparent “response” was just trigger avoidance.

Limitations should also be acknowledged. The study was single-center, open-label, non-randomized, and non-placebo-controlled. Early response times were based on structured patient reports during daily follow-up rather than continuous objective monitoring, which may be influenced by expectancy and reporting bias. Although patients were encouraged to deliberately expose themselves to typical pressure triggers to reduce the likelihood of avoidance-driven apparent improvement, we did not incorporate blinded outcome assessment or uniform repeat objective provocation testing at prespecified timepoints for all participants. Moreover, although our findings strongly support effectiveness, they do not define the optimal long-term dosing strategy, treatment duration, or relapse dynamics after discontinuation, questions that remain relevant across chronic urticaria management (1).

In conclusion, this cohort provides real-life evidence that omalizumab produces a rapid and robust clinical benefit in dermographometer-confirmed, antihistamine-refractory DPU, with most patients achieving complete control within 24–48 hours. These findings support the need for further prospective multicenter studies, ideally controlled, to confirm efficacy, characterize durability, and define the role of standardized pressure-provocation threshold testing as an indispensable objective tool for diagnosis and, pragmatically, a functional “clinical biomarker” for monitoring treatment responsiveness in DPU.

## Data Availability

The raw data supporting the conclusions of this article will be made available by the authors, without undue reservation.
